# Acral Persistent Papular Mucinosis in the United States: Case Series and Literature Review

**DOI:** 10.2196/77714

**Published:** 2025-09-16

**Authors:** Devin Miller, Rachel Manci, Jay Patel, William Guo, Daniel Lozeau, James Briley

**Affiliations:** 1 Department of Dermatology, Stony Brook University Hospital, 1320 Stony Brook Road, Building F Suite 200, Stony Brook, NY, 11790, United States, 1 (631) 444-4200; 2 Lewis Katz School of Medicine at Temple University, Philadelphia, PA, United States

**Keywords:** acral persistent papular mucinosis, dermoscopy, dermatology, lichen myxedematosus, mucin

## Abstract

**Background:**

Acral persistent papular mucinosis (APPM) is a localized variant of lichen myxedematosus (LM) characterized by asymptomatic, flesh-colored papules primarily distributed on the hands and forearms. This chronic dermatosis, distinct from generalized mucinosis due to its lack of systemic involvement, remains underreported in medical literature.

**Objective:**

In this study, we present two cases of APPM to the limited pool of documented cases in the United States, highlighting its emerging recognition.

**Methods:**

This is a case series of two patients presenting with asymptomatic papular eruptions on the hands and wrists, consistent with the typical presentation of APPM. Diagnostic confirmation via biopsy revealed focal cutaneous mucinosis. Comprehensive laboratory evaluations, including serum and urine protein electrophoresis, showed no evidence of underlying gammopathy in either patient.

**Results:**

Treatment modalities for APPM are limited and often ineffective. Unlike other forms of LM, APPM features are confined to skin lesions, posing primarily as a cosmetic concern with a favorable prognosis. Accurate diagnosis of this localized LM is crucial to differentiate it from the more severe, generalized form, scleromyxedema, which can have organ involvement and may become fatal. Notably, while spontaneous resolution is reported in LM, including discrete papular mucinosis, APPM typically persists without resolution even after extended follow-up.

**Conclusions:**

These cases underscore the importance of recognizing APPM and advocating for broader awareness and exploration of its clinical variability, etiology, and management strategies. With increasing recognition, the understanding of APPM can be enhanced, paving the way for optimized management and improved outcomes for affected individuals.

## Introduction

Acral persistent papular mucinosis (APPM) is a chronic, localized subtype of papular mucinosis, also known as lichen myxedematosus (LM). LM is characterized by lichenoid cutaneous manifestations, mucinous deposits, fibroblast proliferation, and dermal fibrosis. APPM is distinct in its localized nature, primarily affecting the extensor surfaces of the distal forearms and hands. Notably, it lacks systemic involvement and is not associated with the thyroid diseases seen in generalized forms [1]. To our knowledge, only six cases have been reported in the United States, with approximately 70 additional cases documented across Europe, North America, South America, and Asia, highlighting the limited available literature [1-5].

Classically, APPM presents as asymptomatic, solitary, white- or flesh-colored papules on the extensor surfaces of the hands, wrists, and dorsal forearms, ranging from 2 to 5 mm in size. These papules contain mucin deposits in the upper reticular dermis and often persist for years [1,6]. Nonetheless, uncommon outliers exist, with APPM-like mucinosis reported on the legs and chest [7-9]. Additionally, pruritic lesions have been reported in isolated cases [2]. These findings challenge the traditional assumption that APPM is an asymptomatic cutaneous condition limited to the forearms and hands. A potential genetic and environmental role has been suggested based on familial occurrences of APPM; however, the etiopathogenesis of the disease has yet to be explored extensively and remains uncertain [2,9].

Herein, we present two cases of APPM, helping to shed light on a condition currently underreported in the medical literature. Consent for the publication of all patient photographs and medical information is provided by the authors, stating that all patients gave consent for their photographs and medical information to be published in print and online versions and with the understanding that this information may be publicly available.

## Case Descriptions

**Figure 1.**
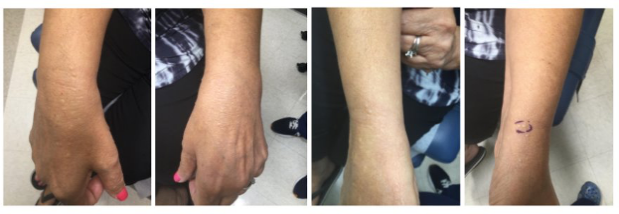
Flesh-colored to slightly yellow firm flat-topped papules on the bilateral dorsal hands and wrists. A biopsy was obtained from the circled lesion on the left dorsal wrist.

Case 1: A 64-year-old female patient with papular eruption on the hands (Figures1  2).

**Figure 2.**
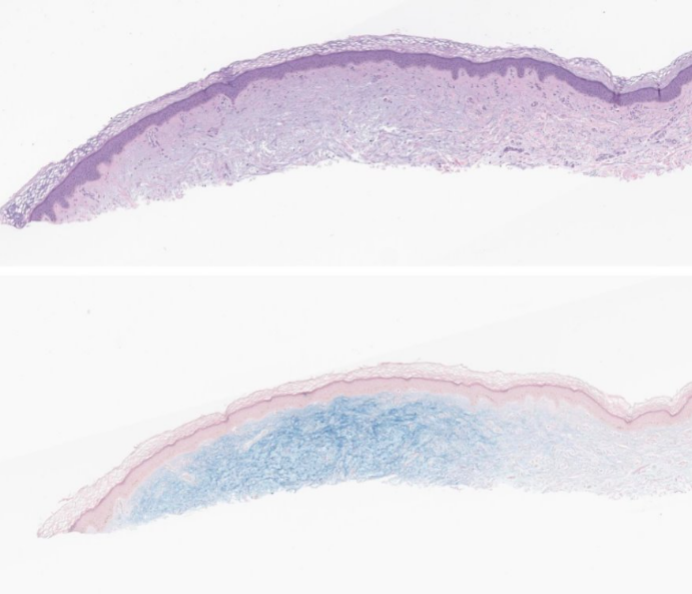
A shave biopsy with H&E (a) revealed deposition of blue-gray mucin within the superficial dermis, highlighted by colloidal iron stain, (b) Original magnification 40X.

A 64-year-old female patient with no significant past medical history presented with a several-year history of an intermittently pruritic papular eruption on both hands. Physical examination revealed multiple superficial flesh-colored papules ranging from 2‐5mm in size on the bilateral dorsal hands, wrists, and distal forearms (Figure 1). Biopsy from the left wrist demonstrated focal cutaneous mucinosis, and a colloidal iron stain confirmed mucin deposition within the superficial dermis (Figure 2). Serum protein electrophoresis and urine protein electrophoresis were negative, aiding in ruling out an underlying monoclonal gammopathy. The patient was diagnosed with APPM and elected to defer any treatment. There has been no progression or spontaneous resolution of her condition to date.

**Figure 3.**
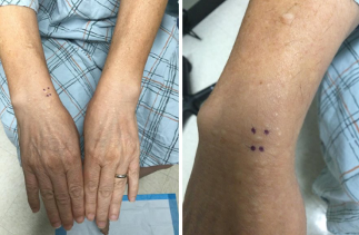
Flesh-colored to slightly yellow spongy papules on the bilateral dorsal hands and wrists. A biopsy was obtained from the circled lesion of the right dorsal wrist.

Case 2: A 67-year-old male patient with papular eruption on the dorsal hands and wrists (Figure 3).

A 67-year-old male patient with no pertinent past medical history presented with a several-year history of stable papular eruptions on the dorsal hands, wrists, and distal forearms. The physical examination revealed focally scattered, flesh-colored, spongy papules measuring 2‐5 mm in size. A biopsy from the right dorsal wrist revealed focal cutaneous mucinosis. Based on the clinicopathological correlation and lack of systemic involvement, a diagnosis of APPM was made. The patient deferred treatment and was subsequently lost to follow-up; to our knowledge, there was no progression or spontaneous resolution of his condition during the observed period.

## Discussion

We present two additional cases of APPM, adding to the six previously reported cases in the United States literature [2]. This may reflect either a rising recognition of the condition or significant underreporting to date.

Notably, both of our patients were predominantly asymptomatic and had no underlying medical conditions, consistent with prior reports suggesting that APPM is not associated with systemic disease or an underlying gammopathy [10]. Unlike other forms of LM, APPM is a skin-limited condition with a favorable prognosis.

Accurate diagnosis of APPM is crucial to differentiate it from generalized LM, scleromyxedema, which can have organ involvement and may be fatal without proper diagnosis and treatment. Diagnostic features of APPM include the presence of ivory to flesh colored papules ranging 2‐5mm in size, female predominance, persisting without spontaneous resolution, and the absence of systemic disease overlap or associated gammopathy [10]. Histologically, APPM is characterized by focal, well-circumscribed mucin in the papillary and mid dermis, sparing the Grenz zone, with the absence or variations of fibroblast proliferation [10]. Unlike other forms of LM, including discrete papular mucinosis, which may resolve spontaneously, APPM generally persists over time, as observed in our cases [2].

A variety of treatment strategies for APPM have been described in the literature. Topical and intralesional corticosteroids have shown minimal to no clinical improvement [2]. Tacrolimus 0.1% ointment has been postulated as a potential treatment option for LM by inhibiting tumor necrosis factor (TNF)-α secretion and transforming growth factor (TGF)-β-induced collagen synthesis, although only a partial response has been reported in the literature [2,9]. Destructive modalities, such as electrofulguration, have demonstrated efficacy in lesion resolution, albeit with mild scarring [2].

In conclusion, our case series highlights the importance of recognizing APPM and adds to the 70 documented cases worldwide, including now eight from the United States (Table 1). As APPM remains an underreported entity in the medical literature, these cases serve to enhance awareness and encourage further exploration into its clinical variability, etiology, genetic predispositions, and optimal management strategies. Importantly, our cases provide additional evidence to support accurate diagnostic approaches that help distinguish APPM from more severe forms of LM, such as scleromyxedema. Proper diagnosis can help prevent unnecessary treatment and testing. Increased recognition of APPM will ultimately enhance understanding of the condition and guide better management, leading to improved outcomes for affected patients.

**Table 1.** Summary of the APPM cases reported from clinics in the US [2,10].

Study name	Author and year	Patient demographics	Clinical features	Histological findings
Acral persistent papular mucinosis: a distinctive dermal mucinosis. Case reported at the meeting of the American Academy of Dermatology, San Antonio, Texas	Berbaum 1987^a^ [11]	N/A^b^	N/A	N/A
Acral persistent papular mucinosis	Fosko 1992 [12]	40-year-old-female	Back of hands, extensor aspect of wrists Developingx1 yr	N/A
Flesh-colored papules on the wrists of a 61-year-old man	Kineston 2004 [13]	61-year-old-male	Back of wrists and hands gradual increase in #x5 yrs	N/A
Acral persistent papular mucinosis	Harris 2004 [10]	55-year-old-female	Back of hands, extensor aspect of wrists and forearms; Increasing in #x5 yrs	Mild epidermal thinning with intact structure; widened dermal collagen spacing. Alcian blue staining revealed defined mucin deposits in upper/mid-reticular dermis, sparing the grenz zone. Hyaluronidase digestion confirmed hyaluronic acid. Scattered fibrocytes and mast cells present [10]
‘Spreading bumps’ on hands of a Native American	Sebastian 2008 [14]	62-year-old-male	Dorsa of hands, wrists and extensor forearms slowly spreading	N/A
Treatment of acral persistent papular mucinosis using an Erbium-YAG^c^ laser	Graves 2015 [15]	60-year-old-female	Dorsal hands	A tissue sample taken from a lesion on the right dorsal hand showed localized mucin accumulation when examined with colloidal iron stain, aligning with features of acral persistent papular mucinosis.
Our manuscript (Case 1)	—^e^	64-year-old-female	Dorsal hands and wrists x several years	A shave biopsy from the left wrist with H&E^d^ (Figure 2) revealed deposition of blue-gray mucin within the superficial dermis, highlighted by colloidal iron stain (Figure 2).
Our manuscript (Case 2)	—^e^	67-year-old-male	Dorsal hands and wrists	A biopsy from the right dorsal wrist revealed focal cutaneous mucinosis.

a No full text was available.

b N/A: not available.

c YAG: Yttrium Aluminum Garnet.

d H&E: Hematoxylin and Eosin.

e Not applicable.

## Abbreviations

APPM: acral persistent papular mucinosis
LM: lichen myxedematosus
